# Wide range of applications for machine-learning prediction models in orthopedic surgical outcome: a systematic review

**DOI:** 10.1080/17453674.2021.1932928

**Published:** 2021-06-10

**Authors:** Paul T Ogink, Olivier Q Groot, Aditya V Karhade, Michiel E R Bongers, F Cumhur Oner, Jorrit-Jan Verlaan, Joseph H Schwab

**Affiliations:** a Department of Orthopedic Surgery, University Medical Center Utrecht – Utrecht University, Utrecht, The Netherlands; b Department of Orthopedic Surgery, Orthopedic Oncology Service, Massachusetts General Hospital – Harvard Medical School, Boston, USA

## Abstract

Background and purpose — Advancements in software and hardware have enabled the rise of clinical prediction models based on machine learning (ML) in orthopedic surgery. Given their growing popularity and their likely implementation in clinical practice we evaluated which outcomes these new models have focused on and what methodologies are being employed.

Material and methods — We performed a systematic search in PubMed, Embase, and Cochrane Library for studies published up to June 18, 2020. Studies reporting on non-ML prediction models or non-orthopedic outcomes were excluded. After screening 7,138 studies, 59 studies reporting on 77 prediction models were included. We extracted data regarding outcome, study design, and reported performance metrics.

Results — Of the 77 identified ML prediction models the most commonly reported outcome domain was medical management (17/77). Spinal surgery was the most commonly involved orthopedic subspecialty (28/77). The most frequently employed algorithm was neural networks (42/77). Median size of datasets was 5,507 (IQR 635–26,364). The median area under the curve (AUC) was 0.80 (IQR 0.73–0.86). Calibration was reported for 26 of the models and 14 provided decision-curve analysis.

Interpretation — ML prediction models have been developed for a wide variety of topics in orthopedics. Topics regarding medical management were the most commonly studied. Heterogeneity between studies is based on study size, algorithm, and time-point of outcome. Calibration and decision-curve analysis were generally poorly reported.

Surgical decision-making in orthopedic surgery involves weighing the benefits of an intervention against its inherent risks. Prognostic scoring tools have been devised to individualize risk prediction and thus improve surgical decision-making (Janssen et al. 2015, Pereira et al. 2016, Shah et al. 2018). Although clinical prediction models are not new, recent advancements in artificial intelligence have created a host of prediction models based on machine learning (ML) (Cabitza et al. 2018).

ML is a branch of artificial intelligence that enables computer algorithms to learn from experience from large datasets without explicit programming. Figure 1 shows 3 commonly employed algorithms. Existing reviews of machine learning studies have provided a broad overview of applications ranging from vision to natural language processing and predictive analytics (Cabitza et al. 2018). To our knowledge, there is no study that has critically assessed the body of studies focused on ML prediction models for surgical outcome in orthopedics. These types of prediction models are most likely the first branch of artificial intelligence to be employed in clinical practice (Staartjes et al. 2020). Therefore, familiarizing practicing orthopedic surgeons with ML’s concepts and the topics these new methods have focused on can optimize their implementation in clinic.

**Figure 1.**
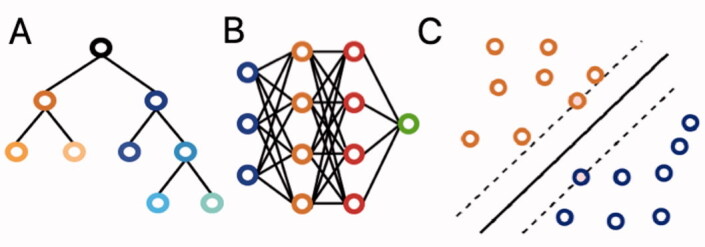
(A) Decision trees are hierarchical structures in which each node performs a test on the input value with the subsequent branches representing the outcomes. Their graphical representation as seen here makes them easy to understand and interpret. However, they are prone to overfitting. (B) Neural networks are based on interconnected nodes. The input features are represented by the first (blue) layer. The designated outcome is represented by the final (green) layer. The middle, hidden layers (blue and orange) base their output on the input they get from prior layers. Neural networks have been around for a long time and offer good discriminative abilities, but interpretation of the relationships between the different layers remains difficult. (C) Support vector machines (SVMs) perform classification by determining the optimal separating hyperplane between datapoints, which maximizes the distance between the 2 closest points of either group. They can be used for both linear and nonlinear relationships. While they remain effective in data with a great number of features, they do not work well in larger datasets.

As such, the purpose of this systematic review is to (1) evaluate which surgical outcomes orthopedic clinical prediction models have focused on, and (2) determine which techniques current prediction models use for development and validation.

## Material and methods

### Systematic literature search

**Figure 2.**
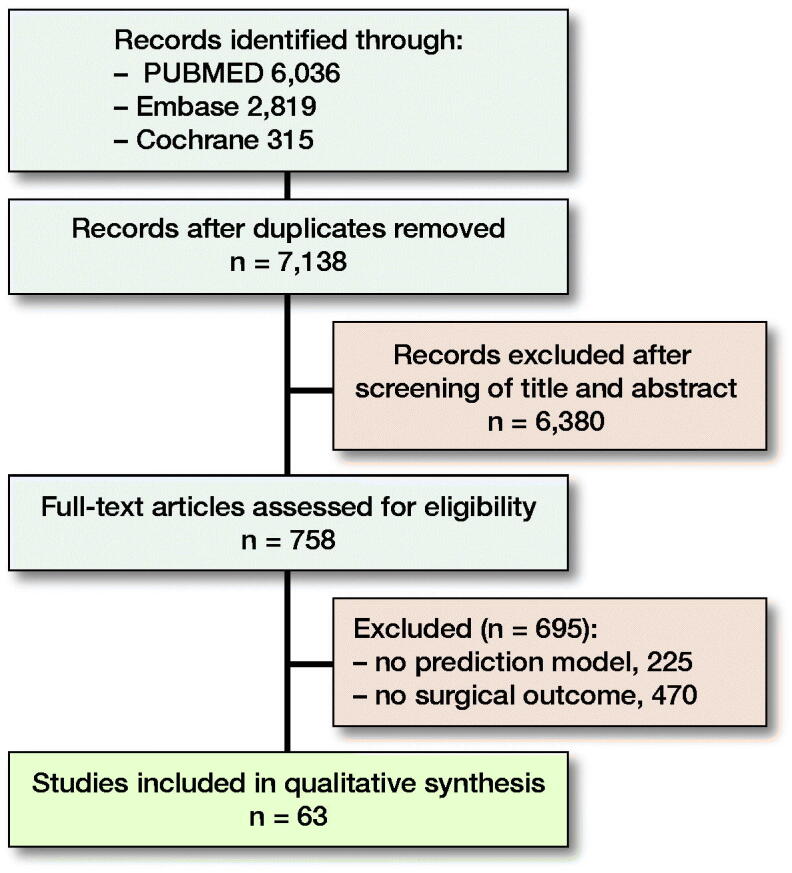
Flowchart of study inclusions and exclusions.

Adhering to the 2009 PRISMA guidelines a systematic search was performed in PubMed, Embase, and the Cochrane Library for articles published up to June 18, 2020. 2 different domains of medical subject headings (MeSH) terms and keywords were combined with “AND” and within the 2 domains the terms were combined with “OR.” The 1st domain included words related to ML and the second domain related to possible orthopedic specialties (Appendix 1, see Supplementary data). Terms were restricted to MeSH, title, abstract, and keywords. Two reviewers (PTO, OQG) independently screened all titles and abstracts for eligible articles based on predefined criteria. Eligible full-text articles were evaluated and cross-referenced for potentially relevant articles not identified by the initial search (Figure 2). Discrepancies between the 2 reviewers were adjudicated by the senior author (JHS).

### Eligibility criteria

Studies reporting on ML-based prediction models addressing orthopedic surgical outcomes were included, as were all intraoperative and postoperative outcomes. The surgical orthopedic population was defined as disorders of the bones, joints, ligaments, tendons, or muscles treated by any type of operation. Excluded were studies (1) that did not include at least 1 ML-based prediction models for surgical outcome (e.g., logistic regression-based models), (2) non-English studies, (3) lack of full text, and (4) non-relevant study types such as animal studies, letters to the editors, and case reports.

### Assessment of methodological quality

Quality assessment was performed based on a modified nine-item Methodological Index for Non-Randomized Studies (MINORS) checklist (Slim et al. 2003). We made it applicable to our systematic review by including disclosure, study aim, input feature, output feature, validation method, dataset distribution, performance metric, and explanation of the used AI model (Langerhuizen et al. 2019). These 9 items were scored on a binary scale: 0 (not reported or unclear) and 1 (reported and adequate).

### Data extraction

Table 1 lists the data we extracted from each study. For this review, 6 main orthopedic surgical outcome domains were identified, consisting of (1) intraoperative complications (e.g., blood transfusion, prolonged operative time), (2) postoperative complications (e.g., venous thromboembolism), (3) survival, (4) patient reported outcome measures (PROMs), (5) medical management (e.g., hospitalization), and (6) other. For studies reporting the performance of multiple ML models, the best performing ML model was used. 13 studies provided multiple models for multiple surgical outcomes; these were extracted separately resulting in more ML models than studies. Only the 2 performance measures AUC and accuracy were extracted as they were most the commonly reported results.

**Table 1.** Data extracted from each study

1	Year of publication
2	First author
3	Disease condition
4	Type of surgery
5	Input feature
6	Number of features in final model
7	Type of outcome
8	Time points of outcome
9	Number of output classes
10	ML algorithm used
11	Number of patients
12	Distribution between training, validation, and test set
13	Validation method
14	AUC and accuracy of model
15	Reporting of calibration and Brier score
16	Decision-curve analysis
17	Digital application of the model

### Study characteristics

After screening of titles and abstracts, 758 full-text articles were assessed for eligibility and ultimately 59 articles were included reporting on 77 ML prediction models (Table 1). Median sample size was 5,818 (IQR 635–26,869). Using the MINORS criteria, all 59 articles were found to be of similar quality. All included a minimum of 8 out of 9 appraisal items (Appendix 2, see Supplementary data).

### Statistics

AUC scores and accuracies in tables are expressed as they were originally reported. For studies that reported multiple results within a single outcome domain (e.g., multiple different postoperative PROMs, each with an independent AUC) averages were taken. The sizes of the training, validation, and test sets are reported as percentages of the total dataset. No meta-analysis was performed because of obvious heterogeneity between studies and in orthopedic applications. However, to summarize the findings in some quantitative form, the median AUC and accuracy of the prediction performance were calculated for all studies.

We used Microsoft Excel (Version 16.31; Microsoft Inc, Redmond, WA, USA) for standardized forms for data extraction and quality assessment, and Mendeley as reference management software.

### Ethics, funding, and potential conflicts of interests

Institutional review board approval was not required for this systematic review. No external funding was received. The authors have no conflicts of interest to declare.

## Results

### Study design

Table 2 lists the characteristics of all included studies. More than half of the 77 models were developed with data from national databases or registries (42) (Table 3). The median number of predictor variables used in the ML model was 10 (IQR 8–15). Models using national data did not include more variables: 10 (IQR 8–13). 68 of the models had a binary distribution of the outcome variable. Most frequently employed algorithms were neural networks (42) and random forests (30). 36 of the neural networks were single-layer, 5 deep learning, and 1 convolutional. The median number of patients used was 5,507 (IQR 635–26,364). Median AUC was 0.80 (IQR 0.73–0.86) and median accuracy was 79% (IQR 75–88). Calibration was reported for 26 of the models and 23 provided Brier scores. Decision-curve analysis was employed in 14 studies. 18 provided a digital application for their prediction model.

**Table 3.** Characteristics of studies (n = 77). Values are count (%) unless otherwise specified

Sample size, median (IQR)	5,818 (635–26,364)
Predictors included in final model, median (IQR) a	10 (8–15)
Outcome domain	
Medical management	17 (22)
Survival	16 (21)
Complication	15 (19)
PROMs	12 (16)
Intraoperative complication	3 (3.9)
Other	14 (18)
Orthopedic subspecialty	
Spine	28 (36)
Arthroplasty	21 (27)
Trauma	13 (17)
Oncology	6 (7.8)
Other	9 (12)
National/Registry database b	42 (55)
Split sample	
70:30	22 (29)
80:20	19 (25)
Other	36 (46)
ML algorithm c	
Neural network	42 (55)
Single layer	36 (47)
Deep learning	6 (8)
Convolutional	1 (1)
Random forest	30 (39)
Support vector machine	19 (25)
Naive Bayes	11 (14)
Stochastic gradient boosting	10 (13)
Performance metric c	
AUC	74 (96)
Accuracy	39 (51)
Brier score	23 (30)
Calibration	26 (34)
Model explanation	
Global	34 (44)
Local	17 (22)
Decision curve analysis	14 (18)
Digital application available	18 (23)

AUC = area under the curve, IQR = interquartile range, ML = machine learning, PROM = patient reported outcome measure.

**^a^** Amount of predictors that were included in the final, best performing machine learning algorithm. In 16% (13/81) this could not be extracted from the study or was unclear.

**^b^** This includes databases such as Surveillance, Epidemiology, and End Results (SEER) or American College of Surgeons National Surgical Quality Improvement Program (ACS NSQIP).

**^c^** Not mutually exclusive.

**Table 2.** Studies evaluating ML models for orthopedic surgical outcome prediction

ABC	D	E	F		G	H	I	J	K	L	M	N	O	P
Intraoperative	complications													
Durand, 2018	SpD	NOS	C, S, H	4	Intraop.	3 d	2	RF, DT	1,029	80	10-FCV	20	0.85	
Huang, 2018	NA	THA, TKA	C, S	7	Intraop.	NA	2	RF, LR	15,187	100	5-FCV	NA	0.84	
Siccoli, 2019	SpS	Decomp.	C	15	Intraop.	45 min	2	RF, XGB, BDT, KNN, ANN, GLM, BGLM	635	70	NA	30	0.54	78
Postoperative	complications													
Arvind, 2018	NA	ACDF	C		Compl.	NA	2	ANN, SVM, RF	20,879	70	5-FCV	30	0.65	
Fatima, 2020	Degen. SO	NOS	C, S	10	Compl.	1 m	2	LR, LASSO	80,610	70	10-FCV	30	0.70	
Gowd, 2019	Shoulder arthritis	TSA	C, S		Compl.	1 m	2	LR, GBM, RF, KNN, DT, NB	17,119	80	CV (nos)	20	0.71	95
Han, 2019	SpP	Sp surg.	C, S	274	Compl.	1 m	2	LR, LASSO	11,04233	70	10-FCV	30	0.70	
Harris, 2018	OA	THA, TKA	C, S	13	Compl.	1 m	2	BR, LASSO	70,569	100	10-FCV	NA	0.70	
Harris, 2019	Nonemergent	THA, TKA	C	Compl.	1 m	2	LASSO	10,7792	100	10-FCV	NA	0.64		
primary														
Hopkins, 2020a	SpP	Posterior fusion	C, S, H		Compl.	NA	NN	4,046	75	CV (nos)	25	0.79		
Karhade, 2020a	SpP	ALIF	C, S	6	Compl.	intra- op.	EPLR, SGB, RF, SVM, NN	1,035	75	CV (nos)	25	0.73		
Kim, 2018a	SpD	NOS	C	12	Compl.	NA	2	ANN, LR	5,818	70	5-FCV	30	0.64	
Kim, 2018b	Degen. SpP	PLIF	C	12	Compl.	NA	2	ANN, LR	22,629	70	NA	30	0.63	
Kukar, 1996	Femur fracture	NOS	C	17	Compl.	24 m	2	Backpropagation	151	70	10-FCV	30	71	
								ANN, NB, KNN, LFC, DT						
				17			5	Semi NB, ANN, NB, KNN, LFC, DT	151	70	10-FCV	30	67	
Pua, 2019	Knee OA	TKA	C		Compl.	6 m	2	LR, RF, GBM	4,026	70	ICVL	30	0.75	
Scheer, 2017	Adult SpD	NOS	C, S, R	20	Compl.	1.5 m	2	RT	557	70	NA	30	0.89	88
Wu, 2016	Lower extrem ities (NOS)	NOS (inclu ding PCEA)	C, S	9	Compl.	NA	2	SVM, LR	195	75	CV (nos)	25	0.93	88
Medical management														
Gabriel, 2019	OA	THA	C	9	Hosp.	≤ 3 d	2	RR, LASSO, RF, MLR	960	67	NA	33	0.76	
Goyal, 2019	SpP	Spinal fusion	C		Non-HD	1 m	2	GLM, NB, ANN, RF, GBM, LDA	59,145	100	10-FCV	NA	0.87	79
Gowd, 2019	Shoulder arthritis	TSA	C, S	Extended LOS	1 m	2	GBM, RF, KNN, DT, NB, LR	17,119	80	CV (nos)	20	0.68	82	
Karhade, 2018b	LDDD	NOS	C	10	Non-HD	NA	2	NN, BPM, BDT, SVM	26,364	80	10-FCV	20	0.82	
Karnuta, 2019	Hip fracture	NOS	C	7	Hosp.	NA	4	NB	98,562	90	10-FCV	10	0.88	77
		NOS	C	7	Cost	NA	3	NB	98,562	90	10-FCV	10	0.89	79
Karnuta, 2020	SpP	Sp. fusionl	C	8	Cost	NA	3	NB	38,070	100	10-FCV	NA	0.88	80
		Sp. fusionl	C	8	LOS	NA	3	NB	38,070	100	10-FCV	NA	0.94	87
		Sp. fusionl	C	8	Non-HD	NA	3	NB	38,070	100	10-FCV	NA	0.91	88
Merrill, 2018	Ankle fracture	ORIF	C	9	Hosp.	3 d	2	Bo, LR	16,501	70	CV (nos)	30	0.76	72
Ogink, 2019b	SpS	Surgery	C	10	Non-HD	NA	2	ANN, SVM, BPM, BDT	28,600	80	10-FCV	20	0.74	
Ogink, 2019a	Degen. SO	Surgery	C	10	Non-HD	NA	2	BPM, ANN, SVM, BDT	9,338	80	10-FCV	20	0.75	
Ottenbacher, 2004	Hip fracture	NOS	C, R	6	Non-HD	80 d	2	ANN, LR	3,708	67	3-FCV	33	0.73	
Ramkumar, 2019	OA	THA	C, H	15	LOS	NA	2	ANN	78,335	100	10-FCV	0.82	75	
		THA	C, H	15	Charges	NA	2	ANN	78,335	100	10-FCV	0.83	76	
		THA	C, H	15	Non-HD	NA	2	ANN	78,335	100	10-FCV	0.79	72	
Siccoli, 2019	SpS	Decomp.	C	15	Hosp.	28 h	2	XGB, RF, BDT KNN, ANN, GLM, BGLM	635	70	NA	30	0.58	77
PROMs														
Azimi, 2014	Lumbar SpS	NOS	C	7	PROM	24 m	2	ANN, LR	168	50	25	25	0.80	97
Fontana, 2018	OA	THA, TKA	C, S, H		PROM	24 m	2	LASSO, RF, SVM	13,719	80	5-FCV	20	0.80	
Huber, 2018	OA	THA, TKA	C		PROM	NA	2	XGB, ANN,	66,356	97	5-FCV	3	0.81	75
KNN, NB, RF,														
MSAENET, LM, LB														
Khan, 2019	DCM	NOS	C	28	PROM	12 m	2	MARS, CT, SVM, PLS, GBoM, GAM, RF, LR	193	75	10-FCV	25	0.78	71
A B C	D	E	F		G	H	I	J	K	L	M	N	O	P
Kumar, 2020	Shoulder pathology	aTSA	C, S	291	PROM	1 y,	2	NN, LM, DT	4,782	67	NA	33	0.86	91
						2–3, 3–5, > 5 y								
		rTSA	C, S	291	PROM	1 y,	2	NN, LM, DT	4,782	67	NA	33	0.88	94
						2–3, 3–5, > 5 y								
Kunze, 2020	OA	THA	C	8	PROM	24 m		RF, SGB, SVM	616	80	CV (nos)	20	0.97	
NN, EPLR														
Lungu, 2015	OA	THA	C	6	PROM	12 m, 24 m	2	RF	265	100	Bootstrap resamping	NA		89
Merali, 2019	DCM	Decomp.	C, S	5	PROM	6 m, 12, 24 m	2	RF	605	70	10-FCV	30	0.72	71
Nwachukwu, 2020	FAI	Hip arthroscopy	C	5	PROM	24 m	2	LR	1,103	100	10-FCV	NA	0.86	
Siccoli, 2019	SpS	Decomp.	C	15	PROM	1.5 m, 3 m	2	BDT, RF, XGB, KNN, ANN, GLM, BGLM	635	70	NA	30	0.86	76
Schwartz, 1997	OA	THA	C	14	PROM	12 m	2	ANN, LR	221	95	LOOCV	5	0.79	
Survival														
Arvind, 2018	NA	ACDF	C		Survival	NA	2	ANN, SVM, RF	20,879	70	5-FCV	30	0.98	
Chen, 2020	Hip fracture	Nos	C, H	11	Survival	NA	2	ANN	10,534	70	15	15	0.93	93
Forsberg, 2011	Bone metastases	Nos	C		Survival	3 m, 12 m	2	BNN	189	90	10-FCV	10	0.84	
Harris, 2018	OA	THA, TKA	C, S	13	Survival	1 m	2	BR, LASSO	70,569	100	10-FCV	NA	0.73	
Harris, 2019	Elective PA	THA, TKA	C		Survival	1 m	2	LASSO	10,7792	100	10-FCV	NA	0.73	
Karhade, 2018c	Spine metastasis	NOS	C	7	Survival	1 m	2	BPM, NN, DT, SVM	1,790	80	10-FCV	20	0.78	
Karhade, 2018a	Spinal chordoma	NOS	C, S	5	Survival	60 m	2	BPM, BDT, SVM, ANN	265	100	10-FCV	0	0.80	
Karhade, 2019d	Spine metastasis	NOS	C	17	Survival	3 m, 12 m	2	SGB, PLR, RF, NN, SVM	732	80	10-FCV	20	0.86	
Kim, 2018a	SpD	NOS	C	12	Survival	NA	2	ANN, LR	5,818	70	5-FCV	30	0.84	69
Kim, 2018b	Various degen. diseases	PLIF	C	12	Survival	NA	2	ANN, LR	22,629	70	NA	30	0.70	60
Lin, 2010	Femur fracture	Various	C, R	11	Survival	12 m	2	ANN	286	70	NA	30	0.95	96
Merrill, 2018	Ankle fracture	ORIF	C	9	Survival	NA	2	Bo, LR	16,501	70	CV (nos)	30	0.74	85
Paulino	Spine	Various	C	9	Survival	1 m,	2	Nomogram, Bo	649	80	5-FCV	20	0.74	75
Pereira, 2016	metastasis	3, 12 m												
Shi, 2013	Femur fracture	DHS	C	9	Survival	12 m	2	ANN, LR	2,150	67	NA	33	0.87	86
Thio, 2020	Extremity metastatsis	NOS	C	15	Survival	3 m, 12 m	2	SGB, RF,SVN, NN, PLR	1,090	80	10-FCV	20	0.86	
Zhang, 2020b	Pertrochanteric fracture	PFNA	C, H	14	Survival	12 m	2	BNN	448	100	10-FCV	NA	0.85	
Other														
Anderson, 2020	ACL rupture	ACL recon struction	C	opioid use	3 m	2	GBM, LR, BNN, RF	10,919	80	CV (nos)	20	0.77		
Azimi, 2015	LDH	MicroDE	C	14	Recurrence	NA	2	ANN, LR	402	50	NA	25	0.83	94
Bevevino, 2014	Calcaneus fracture	Limb salvage	C, R	8	Amputation	NA	2	ANN, LR	155	100	10-FCV	NA	0.80	79
Hopkins, 2020b	SpP	Posterior fusion	C, S, H	177	Read- mission	1 m	2	ANN	23,264	75	Cv (nos)	25	0.81	79
Kalagara, 2018	NA	Lumbar laminectomy	C, S, H	13	Read- mission	1 m	2	GBM	26,869	85	10-FCV	15	0.81	95
Karhade, 2019a	Cervical pathology	ACDF	C, S	10	Sustained opioid use	3m	2	SGB, RF, NN, SVM, EPLR	2,737	80	10-FCV	20	0.81	
Karhade, 2019b	Hip arthritis	THA	C	7	Sustained opioid use	3 m	2	EPLR, SGB, RF, SVM, ANN	5,507	80	10-FCV	200.77		
Karhade, 2019c	LDH	NOS	C	9	Sustained opioid use	6 m	2	EPLR, RF, SGB, ANN, SVM	5,413	80	10-FCV	20	0.81	
Karhade, 2020b	LDH, SpS, SO	Decomp. and/or fusion	C, S	6	Sustained opioid use	3 m	2	EPLR, SGB, RF, SVM, ANN	8,435	80	10-FCV	20	0.70	
Katakam, 2020	Knee OA	TKA	C	9	Sustained opioid use	6 m	2	SGB, RF, SVM, ANN, EPLR	12,542	80	CV (nos)	20	0.76	
Martini, 2020	Degen. SpP	NOS	C, S	30	Readm.	1 m	2	RF	11,150	75	5-FCV	25	0.75	
Merrill, 2018	Ankle fracture	ORIF	C	9	Readm.	1 m	2	Bo, LR	33,504	70	CV (nos)	30	0.70	85
Siccoli, 2019	SpS	Decomp.	C	15	Reope- rations	NA	2	XGB, RF, BDT, KNN, ANN, GLM, BGLM	635	70	NA	30	0.66	69
														
Zhang, 2020a	Low back and lower extrem- ity pain	Thoracic or lumbar surgery	C, S	9	Sustained opioid use	12 m	2	LR, RF, SGB, SVM, NN	19,317	80	NA	20	0.85	

NA = not available

NOS = not otherwise specified

A.Output category

B. First author, year of publication

C. Disease/condition

DCM = degenerative cervical myelopathy

FAI = femoroacetabular impingement

LDDD = lumbar degenerative disc disease

LDH = lumbar disc herniation

SO = spondylolisthesis

SpD = spinal deformity

SpP = spinal patholgy

SpS = spinal stenosis

D. Operation

ACDF = anterior cervical discectomy and fusion

ALIF = anterior lumbar spine fusion

Decomp. = decompression

DHS = dynamic hip screws

MicroDE = microdiscectomy

ORIF = open reduction and internal fixation

PA = primary arthroplasty

PCEA = patient-controlled epidural analgesia

PLIF = posterior lumbar spine fusion

PFNA = proximal femoral nail antirotation

THA = total hip arthroplasty

TKA = total knee arthroplasty

TSA = total shoulder arthroplasty (a = anatomic, r = reverse)

E.Input features

C = clinical

H = hospital-related factors (surgeon volume, hospital volume)

S = surgical

F. Number of features

G. Output

Hosp. = hospitalization

LOS = length of stay

Non-HD = Non-home discharge

Readm. = readmission

H. Output: time points

I.Number of classes

J.Machine learning model. Best performing ML model is in bold.

ANN = artificial neural network

BDT = boosted decision tree

BGLM = Batesian generalized linear models

BNN = Bayesian belief network

Bo = boosting

BPM = Bayes point machine

BR = boosting regression

CHAID = chi-square automatic interaction dector

CT = classification tree

DT = decision tree

EPLR = elastic-net penalized logistic regression

FCM = fuzzy C-means

FIS = fuzzy inference system

GAM = generalized additive models

GBM = gradient boosting machine

GboM = generalized boosted models

GLM = generalized linear models

KNN = K-nearest neighbors

LASSO = least absolute shrinkage and selection operator

LB = logistic boost

LDA = linear discriminant analysis

LFC = lookahead feature construction

LM = linear model

LR = logistic regression

MARS = multivariable adaptive regression splines

MLR = multivariable logistic regression

MSAENET = multi-step elastic-net

NB = naive Bayes

PCA = principal component analysis

PLR = penalized logistic regression

PLS = partial least squares

RF = random forests

RT = random trees

RR = ridge regression

SGB = stochastic gradient boosting

SVM = support vector model

SVR = support vector regression

XGB = extreme gradient boosting

K.Number of patients

L. Size training set (%)

M. Validation method/size

CV (nos) = cross-validation not otherwise specified

FCV = fold cross validation

ICVL = Inner cross-validation loop

LOOCV = leave-one-out cross validation,

N.Size test set (%)

O. Area under the curve (AUC)

P. Accuracy

### Outcome

The most commonly reported outcome domains were medical management (17) and survival (16). Medical management mostly focused on discharge destination (7) and hospitalization (4). The studies on survival all addressed patient survival. 6 survival studies were in orthopedic oncology and 5 in orthopedic trauma. Both medical management and survival had a higher median AUC (0.82 and 0.84 than overall median AUC). Spinal surgery was the most commonly involved subspecialty (28).

## Discussion

Recent years have seen an increasing interest in artificial intelligence and ML in orthopedics (Bini 2018, Jayakumar et al. 2019). With this systematic review we aimed to provide an introduction to the main concepts of developing ML models for orthopedic surgeons and analyze the current application and design of these models in orthopedic surgery. We found a wide range of potential applications ranging from predicting survival in spinal metastases, clinical outcome after shoulder arthroplasty, and hospitalization after hip fracture surgery.

This systematic review has a number of limitations. 1st, due to the relative novelty of this field of research in orthopedic surgery, the variety in study designs renders comparisons and comprehensive quantitative analysis difficult. We therefore opted to perform a qualitative analysis of the current publications. Hopefully, the increasing familiarity with these types of studies will lead to better reporting and open up the possibility to perform quantitative analyses. 2nd, this review is likely influenced by publication bias. ML prediction models with good performance are more likely to be published than models with mediocre or poor performance. This positive publication bias has been shown both in medicine and computational sciences (Boulesteix et al. 2015). The performance measures presented here were therefore likely to be more favorable than those of all developed models. 3rd, despite our efforts to perform a search across multiple online libraries, we have missed a number of studies reporting ML prediction models. Whilst unfortunate, we do no not think these omissions will significantly alter our findings on research topics or most utilized methodology as this review included nearly 60 studies.

This systematic review shows that ML models have been developed for a wide variety of topics across all subspecialties within orthopedics. Perhaps surprisingly, medical management was the most studied domain with the majority of models focusing on readmissions and discharge placement. Both readmissions and discharge delays impose a heavy burden on healthcare costs (Wan et al. 2016). Healthcare expenditure has risen steadily throughout the developed world in recent decades (OECD 2019). While there is enormous variation in healthcare systems, government institutions in virtually all countries have looked at improving medical management to help curb costs (Schwierz 2016). Papanicolas et al. (2018) found activities relating to planning, regulating, and managing health services was a major factor in the difference in healthcare expenditure between the United States and 10 other high-income countries. Shrank et al. (2019) concluded failure of care coordination, leading to unnecessary readmissions among other things, amounts to $78 billion of waste in the United States. To address this problem the Centers for Medicare and Medicaid Services started the Hospital Readmissions Reduction Program in 2012, incentivizing hospitals to lower readmission rates. Knowing in advance which patients are at risk of being readmitted within 30 days after discharge is crucial, which is a possible explanation as to why so many prediction models focus on this topic. Similarly, knowing in advance where patients are likely to be discharged to makes preventing delayed discharge a lot easier than the other interventions tried over the years (Bryan 2010, Ou et al. 2011). Furthermore, the databases available in the studies on medical management appear to be larger, enabling researchers to include more variables and create better performing prediction models. These models are more likely to be published as evidenced by the higher AUC for medical management compared to overall AUC.

Survival was the other commonly studied outcome domain. Accurately estimating remaining life-expectancy is an important feature in medical decision-making in orthopedic oncology (Pereira et al. 2016). In a patient group with only limited life-span remaining, the aim of treatment is to preserve quality of life. Accurate survival estimations can guide decision-making on whether or not to perform surgery and if so, which operative treatment should be opted for (Quinn et al. 2014). With an ageing population and cancer patients surviving longer, the incidence of bone metastases will continue to rise and prediction models will likely play an increasing role in this field (Quinn et al. 2014).

The AAOS Census 2018 showed only 8.3% of orthopedic surgeons’ primary specialty area was the spine, while one-third of the prediction models were linked to spinal surgery (AAOS Department of Clinical Quality and Value 2019). Cost reduction may also be the driving factor in the overrepresentation of spinal surgery prediction models; the economic cost of spinal surgery is large and growing with spinal fusions alone costing $30 billion annually in the United States (Johnson and Seifi 2018). Prediction models could play a role in curbing costs by improving patient selection and surgical decision-making, although this could be said for all other subspecialties. Another possible explanation for the disproportionate number is the overlap with neurosurgery. The neurosurgical field was relatively quicker to use ML to develop prediction models and had developed several models in spinal surgery earlier on (Senders et al. 2018). Finally, the field of prediction models is expanding but still small. A significant proportion of the prediction models are developed by a few research groups that happen to focus on spine surgery. With the field expanding as fast as it is with new prediction models being published every month, we expect the overrepresentation of spine surgery to be temporary in a field in its infancy.

While there is wide variation in study design, certain study design elements are fairly similar across most studies. The most common designs comprise binary outcomes; either a 70:30 or 80:20 split between training and test set; and 10-FCV as method of internal validation. Wide variety exists in study size, time-point of outcome, and choice of ML algorithms. Study size is mostly defined by whether a national database or registry was used for model development. These quality improvement databases offer a large number of datapoints with a variety of variables of a diverse group of hospitals, enabling the creation of prediction models. However, these databases are sometimes flawed by errors and their generalizability is also yet to be assessed (Rolston et al. 2017). External validation remains crucial considering generalizability outside the geographical origin of the database is not ensured (Janssen et al. 2018). Institutional databases offer the advantage of more veracious data, for instance including PROM data, which can extend over longer periods of time, but often lack adequate size.

Which ML algorithm is chosen seems highly random. While studies do list the pros and cons of certain algorithms, no study elaborates on why those algorithms were specifically chosen. A potential reason neural networks and random forests are selected so often is the familiarity of these algorithms. Neural networks have been around for decades, but were limited by lagging computational power (Hopfield 1988). The increase in computational power has led to a significant expansion of what neural networks can process and scientists have been able to build on the work of previous decades (Schmidhuber 2015). Future research should report on multiple ML algorithms and provide the performance measures of all models, thus enabling comparison between different approaches.

Despite the importance of performance metrics, a mere one-third of prediction models included information on calibration, similar to prior studies assessing prediction models in multiple medical domains (Bouwmeester et al. 2012, Heus et al. 2018). Calibration is important to evaluate wehther the model is under- or overestimating the risk regardless of the discriminative abilities. Systematically underestimating risk can lead to undertreatment, while overestimating risk can cause overtreatment (Van Calster and Vickers 2015, Van Calster et al. 2019). To improve the quality of reporting of clinical prediction models, Collins et al. (2015) published the Transparent Reporting of a multivariable prediction model for Individual Prognosis or Diagnosis (TRIPOD) statement. While not tailored for ML prediction models this guideline can provide a framework for researchers to use during development. Hopefully, a more widespread adaptation of the TRIPOD statement can lead to less variation in study designs and better reporting of performance metrics.

Only one-fifth of prediction models have a digital application available. The purpose of prediction models is to aid clinicians and patients in decision-making, which can be achieved only if the models are available for use. Otherwise, predictive analytics based on ML will remain a mere theoretical exercise. Furthermore, researchers should be encouraged to not only provide a digital application of their prediction model, but share their code as well. With a field in its infancy, providing code of more experienced researchers can guide beginning research groups in their endeavors. Additionally, this can greatly increase the small number of external validation studies being performed.

In conclusion, ML prediction models have been developed for a wide variety of topics in orthopedic surgery. Topics regarding medical management and survival were the most commonly studied and spine surgery was the most involved subspecialty. Heterogeneity between studies is mostly based on study size, choice of ML algorithm, and time-point of outcome. Most published prediction models showed fair to good discriminative abilities, while calibration was poorly reported. Future studies should preferably include more multi-institutional, prospective databases and develop multiple models enabling comparison between different ML approaches. Also, important performance measures such as calibration should be reported to evaluate the prediction model accurately.

## Supplementary Material

Supplemental Material

## Supplementary data

Table 2 and appendices 1 and 2 are available as supplementary data in the online version of this article, http://dx.doi.org/10.­1080/17453674.2021.1932928

## APPENDIX 1: Search syntaxes for the PubMed, Embase, and Cochrane databases

*PubMed: June 18, 2020—6,036 hits*

((“Foot”[Mesh] OR “Ankle”[Mesh] OR “Knee Joint”[Mesh] OR “Knee”[Mesh] OR “Ankle Joint”[Mesh] OR “Hip”[Mesh] OR “Hip Joint”[Mesh] OR “Hip Prosthesis”[Mesh] OR “Hip Fractures”[Mesh] OR “Shoulder Joint”[Mesh] OR “Shoulder”[Mesh] OR “Shoulder Fractures”[Mesh] OR “Shoulder Dislocation”[Mesh] OR “Elbow”[Mesh] OR “Elbow Joint”[Mesh] OR “Wrist Joint”[Mesh] OR “Spine”[Mesh] OR “Intervertebral Disc Degeneration”[Mesh] OR “Bone Neoplasms”[Mesh] OR “Arthroplasty”[Mesh] OR “Fractures, Bone”[Mesh] OR “Orthopedics”[Mesh] OR “Foot”[Tiab] OR “Ankle”[Tiab] OR Knee[Tiab] OR Hip[Tiab] OR “Shoulder”[Tiab] OR Elbow[Tiab] OR Wrist[Tiab] OR Spina*[Tiab] OR Spine*[tiab] OR “degenerative disc”[Tiab] OR “Bone Neoplasms”[Tiab] OR Arthroplast*[Tiab] OR Fractur*[Tiab] OR Orthop*[Tiab])) AND (“Artificial Intelligence”[Mesh] OR “Machine Learning”[Mesh] OR “Supervised Machine Learning”[Mesh] OR “Neural Networks Computer”[Mesh] OR “Deep Learning”[Mesh] OR “support vector machine”[MeSH Terms] OR “support vector machine”[All Fields] OR “Support Vector Machine”[Mesh] OR naive bayes[tiab] OR “bayesian learning”[tiab] OR neural network*[tiab] OR “support vector”[tiab] OR support vectors[tiab] OR random forest[tiab] OR “deep learning”[tiab] OR “machine prediction”[tiab] OR “machine intelligence”[tiab] OR “computational intelligence”[tiab] OR “computational learning”[tiab] OR “computer reasoning”[tiab] OR “machine learning”[tiab] OR convolutional network*[tiab] OR “artificial intelligence”[tiab])

*Embase: June 18, 2020—2,819 hits*

(‘foot’/exp/mj OR ‘ankle’/exp/mj OR ‘knee’/exp/mj OR ‘hip’/exp/mj OR ‘hip prosthesis’/exp/mj OR ‘hip fracture’/exp/mj OR ‘shoulder’/exp/mj OR ‘shoulder fracture’/exp/mj OR ‘shoulder dislocation’/exp/mj OR ‘elbow’/exp/mj OR ‘wrist’/exp/mj OR ‘spine’/exp/mj OR ‘intervertebral disk disease’/exp/mj OR ‘bone tumor’/exp/mj OR ‘arthroplasty’/exp/mj OR ‘fracture’/exp/mj OR ‘orthopedic surgery’/exp/mj OR foot:ab, ti OR ankle:ab,ti OR knee:ab,ti OR hip:ab,ti OR shoulder:ab,ti OR spine:ab,ti OR ‘degenerative disc’:ab,ti OR elbow:ab,ti OR wrist:ab,ti OR ‘bone tumor’:ab,ti OR arthroplasty:ab,ti OR fractur:ab,ti OR orthop:ab,ti) AND (‘artificial intelligence’/exp/mj OR ‘machine learning’/exp/mj OR ‘supervised machine learning’/exp/mj OR ‘artificial neural network’/exp/mj OR ‘deep learning’/exp/mj OR ‘support vector machine’/exp/mj OR ‘bayesian learning’/exp/mj OR ‘neural network’:ab,ti OR ‘naive bayes’:ab, ti OR ‘beyesian learning’:ab,ti OR ‘support vector’:ab,ti OR ‘support vectorts’:ab,ti OR ‘random forest’:ab,ti OR ‘deep learning’:ab,ti OR ‘machine prediction’:ab,ti OR ‘machine intelligence’:ab,ti OR ‘computational intelligence’:ab,ti OR ‘computer learning’:ab,ti OR ‘computer reasoning’:ab,ti OR ‘machine learning’:ab,ti OR ‘convolutional network’:ab,ti OR ‘artificial intelligence’:ab,ti)

*Cochrane: June 18, 2020—315 hits*

([mh Foot] OR [mh Knee] OR [mh “Knee Joint”] OR [mh “Ankle Joint”] OR [mh Hip] OR [mh “Hip Joint”] OR [mh “Hip Prosthesis”] OR [mh “Hip Fractures”] OR [mh “Shoulder Dislocation”] OR [mh Elbow] OR [mh “Elbow Joint”] OR [mh “Wrist Joint”] OR [mh Spine] OR [mh “Intervertebral Disk Degeneration”] OR [mh “Bone Neoplasms”] OR [mh Arthroplasty] OR [mh “Fractures, Bone”] OR [mh Orthopedics] OR ((Foot OR Ankle OR Knee OR Hip OR Shoulder OR Elbow OR Wrist OR Spine OR Spina* OR “degenerative disk” OR “Bone Neoplasms” OR Arthroplast* OR Fractur* OR Orthop*):ti,ab,kw)) AND (([mh “Artificial Intelligence”] OR [mh “Machine Learning”] OR [mh “Supervised Machine Learning”] OR [mh “Neural Networks (Computer)”] OR [mh “Deep Learning”] OR [mh “Support Vector Machine”] OR ((“naive bayes” OR “bayesian learning” OR “neural network*” OR “support vector” OR “support vectors” OR “random forest” OR “deep learning” OR “machine prediction” OR “machine intelligence” OR “computational intelligence” OR “computational learning” OR “computer reasoning” OR “machine learning” OR “convolutional network*” OR “artificial intelligence”):ti,ab,kw)))

Supplementary data

## Appendix 2. Critical appraisal of included studies

**Table:**

		Study	Input	Output	Validation	Dataset	Performance	AI
Author, year	Disclosure	aim	feature	feature	method	distribution	metric	model
Anderson, 2020	1	1	1	1	1	1	1	1
Arvind, 2018	1	1	1	1	1	1	1	1
Azimi, 2014	1	1	1	1	1	1	1	1
Azimi, 2015	1	1	1	1	1	1	1	1
Bevevino, 2014	1	1	1	1	1	1	1	1
Chen, 2020	1	1	1	1	1	1	1	1
Durand, 2018	1	1	1	1	1	1	1	1
Fatima, 2020	1	1	1	1	1	1	1	1
Fontana, 2018	1	1	1	1	1	1	1	1
Forsberg, 2011	1	1	1	1	1	1	1	1
Gabriel, 2019	1	1	1	1	1	1	1	1
Gowd, 2019	1	1	1	1	1	1	1	1
Goyal, 2019	1	1	1	1	1	1	1	1
Han, 2019	1	1	1	1	1	1	1	1
Harris, 2018	1	1	1	1	1	1	1	1
Harris, 2019	1	1	1	1	1	1	1	1
Hopkins, 2020a	0	1	1	1	1	1	1	1
Hopkins, 2020b	1	1	1	1	1	1	1	1
Huang, 2018	1	1	1	0	1	1	1	1
Huber, 2018	1	1	0	1	1	1	1	1
Kalagara, 2018	0	1	1	1	1	1	1	1
Karhade, 2018a	1	1	1	1	1	1	1	1
Karhade, 2018b	1	1	1	1	1	1	1	1
Karhade, 2018c	1	1	1	1	1	1	1	1
Karhade, 2019a	1	1	1	1	1	1	1	1
Karhade, 2019b	1	1	1	1	1	1	1	1
Karhade, 2019c	1	1	1	1	1	1	1	1
Karhade, 2019d	1	1	1	1	1	1	1	1
Karhade, 2020a	1	1	1	1	1	1	1	1
Karhade, 2020b	1	1	1	1	1	1	1	1
Karnuta, 2019	1	1	1	1	1	1	1	1
Karnuta, 2020	1	1	1	1	1	1	1	1
Katakam, 2020	1	1	1	1	1	1	1	1
Khan,2019	1	1	1	1	1	1	1	1
Kim, 2018a	1	1	1	1	1	1	1	1
Kim, 2018b	1	1	1	1	1	1	1	1
Kukar, 1996	0	1	1	1	1	1	1	1
Kumar, 2020	1	1	1	1	0	1	1	1
Kunze, 2020	1	1	1	1	1	1	1	1
Lin, 2010	1	1	1	1	1	1	1	1
Lungu, 2015	1	1	1	1	1	1	1	1
Martini, 2020	1	1	1	1	1	1	1	1
Merali, 2019	1	1	1	1	1	1	1	1
Merrill, 2018	1	1	1	1	1	1	1	1
Nwachukwu, 2020	1	1	1	1	1	1	1	1
Ogink, 2019a	1	1	1	1	1	1	1	1
Ogink, 2019b	1	1	1	1	1	1	1	1
Ottenbacher, 2004	1	1	1	1	1	1	1	1
Paulino Pereira, 2016	1	1	1	1	1	1	1	1
Pua, 2019	1	1	1	1	1	1	1	1
Ramkumar, 2019	1	1	1	1	1	1	1	1
Scheer, 2017	1	1	1	1	1	1	1	1
Schwartz, 1997	0	1	1	1	1	1	1	1
Shi, 2013	1	1	1	1	0	1	1	1
Siccoli, 2019	1	1	1	1	1	1	1	1
Thio, 2020	1	1	1	1	1	1	1	1
Wu, 2016	1	1	1	1	1	1	1	1
Zhang, 2020a	1	1	1	1	0	1	1	1
Zhang, 2020b	1	1	1	1	1	1	1	1
