# Supplementary material for: Wide range of applications for machine-learning prediction models in orthopedic surgical outcome: a systematic review
Source: Acta Orthop. 2021 Jun 10;92(5):526–31. doi: 10.1080/17453674.2021.1932928 (PMC8519550; doi:10.1080/17453674.2021.1932928)
Supplement: Supplemental Material [file IORT_A_1932928_SM2925.pdf]

Supplementary data

APPENDIX 1: Search syntaxes for the PubMed, Embase, and Cochrane databases

PubMed: June 18, 2020—6,036 hits

((“Foot”[Mesh] OR “Ankle”[Mesh] OR “Knee Joint”[Mesh] OR “Knee”[Mesh] OR “Ankle Joint”[Mesh] OR “Hip”[Mesh] OR “Hip Joint”[Mesh] OR “Hip Prosthesis”[Mesh] OR “Hip Fractures”[Mesh] OR “Shoulder Joint”[Mesh] OR “Shoulder”[Mesh] OR “Shoulder Fractures”[Mesh] OR “Shoulder Dislocation”[Mesh] OR “Elbow”[Mesh] OR “Elbow Joint”[Mesh] OR “Wrist Joint”[Mesh] OR “Spine”[Mesh] OR “Intervertebral Disc Degeneration”[Mesh] OR “Bone Neoplasms”[Mesh] OR “Arthroplasty”[Mesh] OR “Fractures, Bone”[Mesh] OR “Orthopedics”[Mesh] OR “Foot”[Tiab] OR “Ankle”[Tiab] OR Knee[Tiab] OR Hip[Tiab] OR “Shoulder”[Tiab] OR Elbow[Tiab] OR Wrist[Tiab] OR Spina*[Tiab] OR Spine*[tiab] OR “degenerative disc”[Tiab] OR “Bone Neoplasms”[Tiab] OR Arthroplast*[Tiab] OR Fractur*[Tiab] OR Orthop*[Tiab])) AND (“Artificial Intelligence”[Mesh] OR “Machine Learning”[Mesh] OR “Supervised Machine Learning”[Mesh] OR “Neural Networks Computer”[Mesh] OR “Deep Learning”[Mesh] OR “support vector machine”[MeSH Terms] OR “support vector machine”[All Fields] OR “Support Vector Machine”[Mesh] OR naive bayes[tiab] OR “bayesian learning”[tiab] OR neural network*[tiab] OR “support vector”[tiab] OR support vectors[tiab] OR random forest[tiab] OR “deep learning”[tiab] OR “machine prediction”[tiab] OR “machine intelligence”[tiab] OR “computational intelligence”[tiab] OR “computational learning”[tiab] OR “computer reasoning”[tiab] OR “machine learning”[tiab] OR convolutional network*[tiab] OR “artificial intelligence”[tiab])

Embase: June 18, 2020—2,819 hits

(‘foot’/exp/mj OR ‘ankle’/exp/mj OR ‘knee’/exp/mj OR ‘hip’/exp/mj OR ‘hip prosthesis’/exp/mj OR ‘hip fracture’/exp/mj OR ‘shoulder’/exp/mj OR ‘shoulder fracture’/exp/mj OR ‘shoulder dislocation’/exp/mj OR ‘elbow’/exp/mj OR ‘wrist’/exp/mj OR ‘spine’/exp/mj OR ‘intervertebral disk disease’/exp/mj OR ‘bone tumor’/exp/mj OR ‘arthroplasty’/exp/mj OR ‘fracture’/exp/mj OR ‘orthopedic surgery’/exp/mj OR foot:ab,ti OR ankle:ab,ti OR knee:ab,ti OR hip:ab,ti OR shoulder:ab,ti OR spine:ab,ti OR ‘degenerative disc’:ab,ti OR elbow:ab,ti OR wrist:ab,ti OR ‘bone tumor’:ab,ti OR arthroplasty:ab,ti OR fractur:ab,ti OR orthop:ab,ti) AND (‘artificial intelligence’/exp/mj OR ‘machine learning’/exp/mj OR ‘supervised machine learning’/exp/mj OR ‘artificial neural network’/exp/mj OR ‘deep learning’/exp/mj OR ‘support vector machine’/exp/mj OR ‘bayesian learning’/exp/mj OR ‘neural network’:ab,ti OR ‘naive bayes’:ab,ti OR ‘beyesian learning’:ab,ti OR ‘support vector’:ab,ti OR ‘support vectorts’:ab,ti OR ‘random forest’:ab,ti OR ‘deep learning’:ab,ti OR ‘machine prediction’:ab,ti OR ‘machine intelligence’:ab,ti OR ‘computational intelligence’:ab,ti OR ‘computer learning’:ab,ti OR ‘computer reasoning’:ab,ti OR ‘machine learning’:ab,ti OR ‘convolutional network’:ab,ti OR ‘artificial intelligence’:ab,ti)

Cochrane: June 18, 2020—315 hits

([mh Foot] OR [mh Knee] OR [mh “Knee Joint”] OR [mh “Ankle Joint”] OR [mh Hip] OR [mh “Hip Joint”] OR [mh “Hip Prosthesis”] OR [mh “Hip Fractures”] OR [mh “Shoulder Dislocation”] OR [mh Elbow] OR [mh “Elbow Joint”] OR [mh “Wrist Joint”] OR [mh Spine] OR [mh “Intervertebral Disk Degeneration”] OR [mh “Bone Neoplasms”] OR [mh Arthroplasty] OR [mh “Fractures, Bone”] OR [mh Orthopedics] OR ((Foot OR Ankle OR Knee OR Hip OR Shoulder OR Elbow OR Wrist OR Spine OR Spina* OR “degenerative disk” OR “Bone Neoplasms” OR Arthroplast* OR Fractur* OR Orthop*):ti,ab,kw)) AND (([mh “Artificial Intelligence”] OR [mh “Machine Learning”] OR [mh “Supervised Machine Learning”] OR [mh “Neural Networks (Computer)”] OR [mh “Deep Learning”] OR [mh “Support Vector Machine”] OR ((“naive bayes” OR “bayesian learning” OR “neural network*” OR “support vector” OR “support vectors” OR “random forest” OR “deep learning” OR “machine prediction” OR “machine intelligence” OR “computational intelligence” OR “computational learning” OR “computer reasoning” OR “machine learning” OR “convolutional network*” OR “artificial intelligence”):ti,ab,kw)))

Appendix 2. Critical appraisal of included studies

Author, year	Disclosure	Study aim	Input feature	Output feature	Validation method	Dataset distribution	Performance metric	AI model
Anderson, 2020	1	1	1	1	1	1	1	1
Arvind, 2018	1	1	1	1	1	1	1	1
Azimi, 2014	1	1	1	1	1	1	1	1
Azimi, 2015	1	1	1	1	1	1	1	1
Bevevino, 2014	1	1	1	1	1	1	1	1
Chen, 2020	1	1	1	1	1	1	1	1
Durand, 2018	1	1	1	1	1	1	1	1
Fatima, 2020	1	1	1	1	1	1	1	1
Fontana, 2018	1	1	1	1	1	1	1	1
Forsberg, 2011	1	1	1	1	1	1	1	1
Gabriel, 2019	1	1	1	1	1	1	1	1
Gowd, 2019	1	1	1	1	1	1	1	1
Goyal, 2019	1	1	1	1	1	1	1	1
Han, 2019	1	1	1	1	1	1	1	1
Harris, 2018	1	1	1	1	1	1	1	1
Harris, 2019	1	1	1	1	1	1	1	1
Hopkins, 2020a	0	1	1	1	1	1	1	1
Hopkins, 2020b	1	1	1	1	1	1	1	1
Huang, 2018	1	1	1	0	1	1	1	1
Huber, 2018	1	1	0	1	1	1	1	1
Kalagara, 2018	0	1	1	1	1	1	1	1
Karhade, 2018a	1	1	1	1	1	1	1	1
Karhade, 2018b	1	1	1	1	1	1	1	1
Karhade, 2018c	1	1	1	1	1	1	1	1
Karhade, 2019a	1	1	1	1	1	1	1	1
Karhade, 2019b	1	1	1	1	1	1	1	1
Karhade, 2019c	1	1	1	1	1	1	1	1
Karhade, 2019d	1	1	1	1	1	1	1	1
Karhade, 2020a	1	1	1	1	1	1	1	1
Karhade, 2020b	1	1	1	1	1	1	1	1
Karnuta, 2019	1	1	1	1	1	1	1	1
Karnuta, 2020	1	1	1	1	1	1	1	1
Katakam, 2020	1	1	1	1	1	1	1	1
Khan,2019	1	1	1	1	1	1	1	1
Kim, 2018a	1	1	1	1	1	1	1	1
Kim, 2018b	1	1	1	1	1	1	1	1
Kukar, 1996	0	1	1	1	1	1	1	1
Kumar, 2020	1	1	1	1	0	1	1	1
Kunze, 2020	1	1	1	1	1	1	1	1
Lin, 2010	1	1	1	1	1	1	1	1
Lungu, 2015	1	1	1	1	1	1	1	1
Martini, 2020	1	1	1	1	1	1	1	1
Merali, 2019	1	1	1	1	1	1	1	1
Merrill, 2018	1	1	1	1	1	1	1	1
Nwachukwu, 2020	1	1	1	1	1	1	1	1
Ogink, 2019a	1	1	1	1	1	1	1	1
Ogink, 2019b	1	1	1	1	1	1	1	1
Ottenbacher, 2004	1	1	1	1	1	1	1	1
Paulino Pereira, 2016	1	1	1	1	1	1	1	1
Pua, 2019	1	1	1	1	1	1	1	1
Ramkumar, 2019	1	1	1	1	1	1	1	1
Scheer, 2017	1	1	1	1	1	1	1	1
Schwartz, 1997	0	1	1	1	1	1	1	1
Shi, 2013	1	1	1	1	0	1	1	1
Siccoli, 2019	1	1	1	1	1	1	1	1
Thio, 2020	1	1	1	1	1	1	1	1
Wu, 2016	1	1	1	1	1	1	1	1
Zhang, 2020a	1	1	1	1	0	1	1	1
Zhang, 2020b	1	1	1	1	1	1	1	1

Table 2. Studies evaluating ML models for orthopedic surgical outcome prediction

A B C	D	E	F		G	H	I	J	K	L	M	N	O	P
**Intraoperative complications**														
Durand, 2018	SpD	NOS	C, S, H	4	Intraop.	3 d	2	**RF**, DT	1,029	80	10-FCV	20	0.85	
Huang, 2018	NA	THA, TKA	C, S	7	Intraop.	NA	2	**RF**, LR	15,187	100	5-FCV	NA	0.84	
Siccoli, 2019	SpS	Decomp.	C	15	Intraop.	45 min	2	**RF**, XGB, BDT, KNN, ANN, GLM, BGLM	635	70	NA	30	0.54	78
**Postoperative complications**														
Arvind, 2018	NA	ACDF	C		Compl.	NA	2	**ANN**, SVM, RF	20,879	70	5-FCV	30	0.65	
Fatima, 2020	Degen. SO	NOS	C, S	10	Compl.	1 m	2	**LR**, LASSO	80,610	70	10-FCV	30	0.70	
Gowd, 2019	Shoulder arthritis	TSA	C, S		Compl.	1 m	2	**LR**, GBM, RF, KNN, DT, NB	17,119	80	CV (nos)	20	0.71	95
Han, 2019	SpP	Sp surg.	C, S	274	Compl.	1 m	2	**LR**, LASSO	11,04233	70	10-FCV	30	0.70	
Harris, 2018	OA	THA, TKA	C, S	13	Compl.	1 m	2	**BR**, LASSO	70,569	100	10-FCV	NA	0.70	
Harris, 2019	Nonemergent primary	THA, TKA	C		Compl.	1 m	2	**LASSO**	10,7792	100	10-FCV	NA	0.64	
Hopkins, 2020a	SpP	Posterior fusion	C, S, H		Compl.	NA		**NN**	4,046	75	CV (nos)	25	0.79	
Karhade, 2020a	SpP	ALIF	C, S	6	Compl.	intra-op.		**EPLR**, SGB, RF, SVM, NN	1,035	75	CV (nos)	25	0.73	
Kim, 2018a	SpD	NOS	C	12	Compl.	NA	2	**ANN**, LR	5,818	70	5-FCV	30	0.64	
Kim, 2018b	Degen. SpP	PLIF	C	12	Compl.	NA	2	**ANN**, LR	22,629	70	NA	30	0.63	
Kukar, 1996	Femur fracture	NOS	C	17	Compl.	24 m	2	**Backpropagation** ANN, NB, KNN, LFC, DT	151	70	10-FCV	30		71
				17			5	**Semi NB**, ANN, NB, KNN, LFC, DT	151	70	10-FCV	30		67
Pua, 2019	Knee OA	TKA	C		Compl.	6 m	2	**LR**, RF, GBM	4,026	70	ICVL	30	0.75	
Scheer, 2017	Adult SpD	NOS	C, S, R	20	Compl.	1.5 m	2	**RT**	557	70	NA	30	0.89	88
Wu, 2016	Lower extremities (NOS)	NOS (including PCEA)	C, S	9	Compl.	NA	2	**SVM**, LR	195	75	CV (nos)	25	0.93	88
**Medical management**														
Gabriel, 2019	OA	THA	C	9	Hosp.	≤ 3 d	2	**RR**, LASSO, RF, MLR	960	67	NA	33	0.76	
Goyal, 2019	SpP	Spinal fusion	C		Non-HD	1 m	2	**GLM**, NB, ANN, RF, GBM, LDA	59,145	100	10-FCV	NA	0.87	79
Gowd, 2019	Shoulder arthritis	TSA	C, S		Extended LOS	1 m	2	**GBM**, RF, KNN, DT, NB, LR	17,119	80	CV (nos)	20	0.68	82
Karhade, 2018b	LDDD	NOS	C	10	Non-HD	NA	2	**NN**, BPM, BDT, SVM	26,364	80	10-FCV	20	0.82	
Karnuta, 2019	Hip fracture	NOS	C	7	Hosp.	NA	4	**NB**	98,562	90	10-FCV	10	0.88	77
		NOS	C	7	Cost	NA	3	**NB**	98,562	90	10-FCV	10	0.89	79
Karnuta, 2020	SpP	Sp. fusionl	C	8	Cost	NA	3	**NB**	38,070	100	10-FCV	NA	0.88	80
		Sp. fusionl	C	8	LOS	NA	3	**NB**	38,070	100	10-FCV	NA	0.94	87
		Sp. fusionl	C	8	Non-HD	NA	3	**NB**	38,070	100	10-FCV	NA	0.91	88
Merrill, 2018	Ankle fracture	ORIF	C	9	Hosp.	3 d	2	**Bo**, LR	16,501	70	CV (nos)	30	0.76	72
Ogink, 2019b	SpS	Surgery	C	10	Non-HD	NA	2	**ANN**, SVM, BPM, BDT	28,600	80	10-FCV	20	0.74	
Ogink, 2019a	Degen. SO	Surgery	C	10	Non-HD	NA	2	**BPM**, ANN, SVM, BDT	9,338	80	10-FCV	20	0.75	
Ottenbacher, 2004	Hip fracture	NOS	C, R	6	Non-HD	80 d	2	**ANN**, LR	3,708	67	3-FCV	33	0.73	
Ramkumar, 2019	OA	THA	C, H	15	LOS	NA	2	**ANN**	78,335	100	10-FCV		0.82	75
		THA	C, H	15	Charges	NA	2	**ANN**	78,335	100	10-FCV		0.83	76
		THA	C, H	15	Non-HD	NA	2	**ANN**	78,335	100	10-FCV		0.79	72
Siccoli, 2019	SpS	Decomp.	C	15	Hosp.	28 h	2	**XGB**, RF, BDT KNN, ANN, GLM, BGLM	635	70	NA	30	0.58	77
**PROMs**														
Azimi, 2014	Lumbar SpS	NOS	C	7	PROM	24 m	2	**ANN**, LR	168	50	25	25	0.80	97
Fontana, 2018	OA	THA, TKA	C, S, H		PROM	24 m	2	**LASSO**, RF, SVM	13,719	80	5-FCV	20	0.80	
Huber, 2018	OA	THA, TKA	C		PROM	NA	2	**XGB**, ANN, KNN, NB , RF, MSAENET, LM, LB	66,356	97	5-FCV	3	0.81	75
Khan, 2019	DCM	NOS	C	28	PROM	12 m	2	**MARS**, CT, SVM, PLS, GBoM, GAM, RF, LR	193	75	10-FCV	25	0.78	71

A	B	C	D	E	F		G	H	I	J	K	L	M	N	O	P
		Kumar, 2020	Shoulder pathology	aTSA	C, S	291	PROM	1 y, 2–3, 3–5, > 5 y	2	**NN**, LM, DT	4,782	67	NA	33	0.86	91
				rTSA	C, S	291	PROM	1 y, 2–3, 3–5, > 5 y	2	**NN**, LM, DT	4,782	67	NA	33	0.88	94
		Kunze, 2020	OA	THA	C	8	PROM	24 m		RF, SGB, SVM NN, EPLR	616	80	CV (nos)	20	0.97	
		Lungu, 2015	OA	THA	C	6	PROM	12 m, 24 m	2	**RF**	265	100	bootstrap resamping	NA		89
		Merali, 2019	DCM	Decomp.	C, S	5	PROM	6 m, 12, 24 m	2	**RF**	605	70	10-FCV	30	0.72	71
		Nwachukwu, 2020	FAI	Hip arthroscopy	C	5	PROM	24 m	2	**LR**	1,103	100	10-FCV	NA	0.86	
		Siccoli, 2019	SpS	Decomp.	C	15	PROM	1.5 m, 3 m	2	**BDT**, RF, XGB, KNN, ANN, GLM, BGLM	635	70	NA	30	0.86	76
		Schwartz, 1997	OA	THA	C	14	PROM	12 m	2	**ANN**, LR	221	95	LOOCV	5	0.79	
**Survival**																
		Arvind, 2018	NA	ACDF	C		Survival	NA	2	**ANN**, SVM, RF	20,879	70	5-FCV	30	0.98	
		Chen, 2020	Hip fracture	Nos	C, H	11	Survival	NA	2	**ANN**	10,534	70	15	15	0.93	93
		Forsberg, 2011	Bone metastases	Nos	C		Survival	3 m, 12 m	2	**BNN**	189	90	10-FCV	10	0.84	
		Harris, 2018	OA	THA, TKA	C, S	13	Survival	1 m	2	**BR**, LASSO	70,569	100	10-FCV	NA	0.73	
		Harris, 2019	Elective PA	THA, TKA	C		Survival	1 m	2	**LASSO**	10,7792	100	10-FCV	NA	0.73	
		Karhade, 2018c	Spine metastasis	NOS	C	7	Survival	1 m	2	**BPM**, NN, DT, SVM	1,790	80	10-FCV	20	0.78	
		Karhade, 2018a	Spinal chordoma	NOS	C, S	5	Survival	60 m	2	**BPM**, BDT, SVM, ANN	265	100	10-FCV	0	0.80	
		Karhade, 2019d	Spine metastasis	NOS	C	17	Survival	3 m, 12 m	2	**SGB**, PLR, RF, NN, SVM	732	80	10-FCV	20	0.86	
		Kim, 2018a	SpD	NOS	C	12	Survival	NA	2	**ANN**, LR	5,818	70	5-FCV	30	0.84	69
		Kim, 2018b	Various degen. diseases	PLIF	C	12	Survival	NA	2	**ANN**, LR	22,629	70	NA	30	0.70	60
		Lin, 2010	Femur fracture	Various	C, R	11	Survival	12 m	2	**ANN**	286	70	NA	30	0.95	96
		Merrill, 2018	Ankle fracture	ORIF	C	9	Survival	NA	2	**Bo**, LR	16,501	70	CV (nos)	30	0.74	85
		Paulino Pereira, 2016	Spine metastasis	Various	C	9	Survival	1 m, 3, 12 m	2	**Nomogram**, Bo	649	80	5-FCV	20	0.74	75
		Shi, 2013	Femur fracture	DHS	C	9	Survival	12 m	2	**ANN**, LR	2,150	67	NA	33	0.87	86
		Thio, 2020	Extremity metastatsis	NOS	C	15	Survival	3 m, 12 m	2	**SGB**, RF,SVN, NN, PLR	1,090	80	10-FCV	20	0.86	
		Zhang, 2020b	Pertrochanteric fracture	PFNA	C, H	14	Survival	12 m	2	**BNN**	448	100	10-FCV	NA	0.85	
**Other**																
		Anderson, 2020	ACL rupture	ACL reconstruction	C		Sustained opioid use	3 m	2	**GBM**, LR, BNN, RF	10,919	80	CV (nos)	20	0.77	
		Azimi, 2015	LDH	MicroDE	C	14	Recurrence	NA	2	**ANN**, LR	402	50	NA	25	0.83	94
		Bevevino, 2014	Calcaneus fracture	Limb salvage	C, R	8	Amputation	NA	2	**ANN**, LR	155	100	10-FCV	NA	0.80	79
		Hopkins, 2020b	SpP	Posterior fusion	C, S, H	177	Readmission	1 m	2	**ANN**	23,264	75	Cv (nos)	25	0.81	79
		Kalagara, 2018	NA	Lumbar laminectomy	C, S, H	13	Readmission	1 m	2	**GBM**	26,869	85	10-FCV	15	0.81	95
		Karhade, 2019a	Cervical pathology	ACDF	C, S	10	Sustained opioid use	3m	2	**SGB**, RF, NN, SVM, EPLR	2,737	80	10-FCV	20	0.81	
		Karhade, 2019b	Hip arthritis	THA	C	7	Sustained opioid use	3 m	2	**EPLR**, SGB, RF, SVM, ANN	5,507	80	10-FCV	20	0.77	
		Karhade, 2019c	LDH	NOS	C	9	Sustained opioid use	6 m	2	**EPLR**, RF, SGB, ANN, SVM	5,413	80	10-FCV	20	0.81	
		Karhade, 2020b	LDH, SpS, SO	Decomp. and/or fusion	C, S	6	Sustained opioid use	3 m	2	**EPLR**, SGB, RF, SVM, ANN	8,435	80	10-FCV	20	0.70	
		Katakam, 2020	Knee OA	TKA	C	9	Sustained opioid use	6 m	2	**SGB**, RF, SVM, ANN, EPLR	12,542	80	CV (nos)	20	0.76	
		Martini, 2020	Degen. SpP	NOS	C, S	30	Readm.	1 m	2	**RF**	11,150	75	5-FCV	25	0.75	
		Merrill, 2018	Ankle fracture	ORIF	C	9	Readm.	1 m	2	**Bo**, LR	33,504	70	CV (nos)	30	0.70	85
		Siccoli, 2019	SpS	Decomp.	C	15	Reoperations	NA	2	**XGB**, RF, BDT, KNN, ANN, GLM, BGLM	635	70	NA	30	0.66	69
		Zhang, 2020a	Low back and lower extremity pain	Thoracic or lumbar surgery	C, S	9	Sustained opioid use	12 m	2	**LR**, RF, SGB, SVM, NN	19,317	80	NA	20	0.85	

NA = not available
NOS = not otherwise specified
A. Output category
B. First author, year of publication
C. Disease/condition
DCM = degenerative cervical myelopathy
FAI = femoroacetabular impingement
LDDD = lumbar degenerative disc disease
LDH = lumbar disc herniation
SO = spondylolisthesis
SpD = spinal deformity
SpP = spinal patholgy
SpS = spinal stenosis
D. Operation
ACDF = anterior cervical discectomy and fusion
ALIF = anterior lumbar spine fusion
Decomp. = decompression
DHS = dynamic hip screws
MicroDE = microdiscectomy
ORIF = open reduction and internal fixation
PA = primary arthroplasty
PCEA = patient-controlled epidural analgesia
PLIF = posterior lumbar spine fusion
PFNA = proximal femoral nail antirotation
THA = total hip arthroplasty
TKA = total knee arthroplasty
TSA = total shoulder arthroplasty (a = anatomic, r = reverse)
E. Input features
C = clinical
H = hospital-related factors (surgeon volume, hospital volume)
S = surgical
F. Number of features
G. Output
Hosp. = hospitalization
LOS = length of stay
Non-HD = Non-home discharge
Readm. = readmission
H. Output: time points
I. Number of classes
J. Machine learning model. Best performing ML model is in bold.
ANN = artificial neural network
BDT = boosted decision tree
BGLM = Batesian generalized linear models
BNN = Bayesian belief network
Bo = boosting
BPM = Bayes point machine
BR = boosting regression
CHAID = chi-square automatic interaction dector
CT = classification tree
DT = decision tree
EPLR = elastic-net penalized logistic regression
FCM = fuzzy C-means
FIS = fuzzy inference system
GAM = generalized additive models
GBM = gradient boosting machine
GboM = generalized boosted models
GLM = generalized linear models
KNN = K-nearest neighbors
LASSO = least absolute shrinkage and selection operator
LB = logistic boost
LDA = linear discriminant analysis
LFC = lookahead feature construction
LM = linear model
LR = logistic regression
MARS = multivariable adaptive regression splines
MLR = multivariable logistic regression
MSAENET = multi-step elastic-net
NB = naive Bayes
PCA = principal component analysis
PLR = penalized logistic regression
PLS = partial least squares
RF = random forests
RT = random trees
RR = ridge regression
SGB = stochastic gradient boosting
SVM = support vector model
SVR = support vector regression
XGB = extreme gradient boosting
K. Number of patients
L. Size training set (%)
M. Validation method/size
CV (nos) = cross-validation not otherwise specified
FCV = fold cross validation
ICVL = Inner cross-validation loop
LOOCV = leave-one-out cross validation,
N. Size test set (%)
O. Area under the curve (AUC)
P. Accuracy

Anderson A B, Grazal C F, Balazs G C, Potter B K, Dickens J F, Forsberg J A. Can predictive modeling tools identify patients at high risk of prolonged opioid use after ACL reconstruction? Clin Orthop Relat Res 2020; 478(7): 00-1618.

Arvind V, Kim J S, Oermann E K, Kaji D, Cho S K. Predicting surgical complications in adult patients undergoing anterior cervical discectomy and fusion using machine learning. Neurospine 2018; 15(4): 329-37.

Azimi P, Benzel E C, Shahzadi S, Azhari S, Mohammadi H R. Use of artificial neural networks to predict surgical satisfaction in patients with lumbar spinal canal stenosis. J Neurosurg Spine 2014; 20(3): 300-5.

Azimi P, Mohammadi H R, Benzel E C, Shahzadi S, Azhari S. Use of artificial neural networks to predict recurrent lumbar disk herniation. J Spinal Disord Tech 2015; 28(3): E161-5.

Bevevino A J, Dickens J F, Potter B K, Dworak T, Gordon W, Forsberg J A. A model to predict limb salvage in severe combat-related open calcaneus fractures. Clin Orthop Relat Res 2014; 472(10): 3002–9.

Chen C Y, Chen Y F, Chen H Y, Hung C T, Shi H Y. Artificial neural network and cox regression models for predicting mortality after hip fracture surgery: A population-based comparison. Medicina (Kaunas) 2020; 56(5): 243.

Durand W M, DePasse J M, Daniels A H. Predictive modeling for blood transfusion after adult spinal deformity surgery: A tree-based machine learning approach. Spine (Phila Pa 1976) 2018; 43(15): 1058-66.

Fatima N, Zheng H, Massaad E, Hadzipasic M, Shankar G M, Shin J H. Development and validation of machine learning algorithms for predicting adverse events after surgery for lumbar degenerative spondylolisthesis. World Neurosurg 2020; 140: 627-41.

Fontana M A, Lyman S, Sarker G K, Padgett D E, MacLean C H. Can machine learning algorithms predict which patients will achieve minimally clinically important differences from total joint arthroplasty? Clin Orthop Relat Res 2019; 477(6): 1267-79.

Forsberg J A, Eberhardt J, Boland P J, Wedin R, Healey J H. Estimating survival in patients with operable skeletal metastases: an application of a bayesian belief network. PLoS One 2011; 6(5): e19956.

Gabriel R A, Sharma B S, Doan C N, Jiang X, Schmidt U H, Vaida F. A predictive model for determining patients not requiring prolonged hospital length of stay after elective primary total hip arthroplasty. Anesth Analg 2019; 129(1): 43-50.

Gowd A K, Agarwalla A, Amin N H, Romeo A A, Nicholson G P, Verma N N, Liu J N. Construct validation of machine learning in the prediction of short-term postoperative complications following total shoulder arthroplasty. J Shoulder Elbow Surg 2019; 28(12): e410-e421.

Goyal A, Ngufor C, Kerezoudis P, McCutcheon B, Storlie C, Bydon M. Can machine learning algorithms accurately predict discharge to nonhome facility and early unplanned readmissions following spinal fusion? Analysis of a national surgical registry. J Neurosurg Spine 2019: 1-11. Online ahead of print.

Han S S, Azad T D, Suarez P A, Ratliff J K. A machine learning approach for predictive models of adverse events following spine surgery. Spine J 2019; 19(11): 1772-81.

Harris A H, Kuo A C, Bowe T, Gupta S, Nordin D, Giori N J. Prediction models for 30-day mortality and complications after total knee and hip arthroplasties for Veteran Health Administration patients with osteoarthritis. J Arthroplasty 2018; 33(5): 1539-45.

Harris A H S, Kuo A C, Weng Y, Trickey A W, Bowe T, Giori N J. Can machine learning methods produce accurate and easy-to-use prediction models of 30-day complications and mortality after knee or hip arthroplasty? Clin Orthop Relat Res 2019; 477(2): 452-60.

Hopkins B S, Mazmudar A, Driscoll C, Svet M, Goergen J, Kelsten M, Shlobin NA, Kesavabhotla K, Smith Z A, Dahdaleh N S. Using artificial intelligence (AI) to predict postoperative surgical site infection: A retrospective cohort of 4046 posterior spinal fusions. Clin Neurol Neurosurg 2020a; 192: 105718.

Hopkins B S, Yamaguchi J T, Garcia R, Kesavabhotla K, Weiss H, Hsu W K, Smith Z A, Dahdaleh N S. Using machine learning to predict 30-day readmissions after posterior lumbar fusion: An NSQIP study involving 23,264 patients. J Neurosurg Spine 2020b; 32(3): 399-406.

Huang Z, Huang C, Xie J, Ma J, Cao G, Huang Q, Shen B, Byers Kraus V, Pei F. Analysis of a large data set to identify predictors of blood transfusion in primary total hip and knee arthroplasty. Transfusion 2018; 58(8): 1855-62.

Huber M, Kurz C, Leidl R. Predicting patient-reported outcomes following hip and knee replacement surgery using supervised machine learning. BMC Med Inform Decis Mak 2019;1 9(1): 3.

Kalagara S, Eltorai A E M, Durand W M, DePasse J M, Daniels A H. Machine learning modeling for predicting hospital readmission following lumbar laminectomy. J Neurosurg Spine 2018; 30(3): 344-52.

Karhade A V, Thio Q, Ogink P, Kim J, Lozano-Calderon S, Raskin K, Schwab J H. Development of machine learning algorithms for prediction of 5-year spinal chordoma survival. World Neurosurg 2018a; 119(0): e842–7.

Karhade A V, Ogink P, Thio Q, Broekman M, Cha T, Gormley W B, Hershman S, Peul W C, Bono C M, Schwab J H. Development of machine learning algorithms for prediction of discharge disposition after elective inpatient surgery for lumbar degenerative disc disorders. Neurosurg Focus 2018b; 45(5): E6.

Karhade A V, Thio Q C B S, Ogink P T, Shah A A, Bono C M, Oh K S, Saylor P J, Schoenfeld A J, Shin J H, Harris M B, Schwab J H. Development of machine learning algorithms for prediction of 30-day mortality after surgery for spinal metastasis. Neurosurgery 2018c; 85(1): E83-E91.

Karhade A V, Ogink P T, Thio Q C B S, Broekman M L D, Cha T D, Hershman S H, Mao J, Peul W C, Schoenfeld A J, Bono C M, Schwab J H. Machine learning for prediction of sustained opioid prescription after anterior cervical discectomy and fusion. Spine J 2019a; 19(6): 976-83.

Karhade A V., Schwab J H, Bedair H S. Development of machine learning algorithms for prediction of sustained postoperative opioid prescriptions after total hip arthroplasty. J Arthroplasty 2019b; 34(10): 2272-7.e1.

Karhade A V, Ogink P T, Thio Q C B S, Cha T D, Gormley W B, Hershman S H, Smith T R, Mao J, Schoenfeld A J, Bono C M, Schwab J H. Development of machine learning algorithms for prediction of prolonged opioid prescription after surgery for lumbar disc herniation. Spine J 2019c; 19(11): 1764-71.

Karhade A V, Thio Q C B S, Ogink P T, Bono C M, Ferrone M L, Oh K S, Saylor P J, Schoenfeld A J, Shin J H, Harris M B, Schwab J H. Predicting 90-day and 1-year mortality in spinal metastatic disease: development and internal validation. Neurosurgery 2019d; 85(4): E671-81.

Karhade A V, Bongers M E R, Groot O Q, Cha T D, Doorly T P, Fogel H A, Hershman S H, Tobert D G, Srivastava S D, Bono C M, Kang J D, Harris M B, Schwab J H. Development of machine learning and natural language processing algorithms for preoperative prediction and automated identification of intraoperative vascular injury in anterior lumbar spine surgery. Spine J 2020a; S1529-9430(20)30135-2. Online ahead of print.

Karhade A V, Cha T D, Fogel H A, Hershman S H, Tobert D G, Schoenfeld A J, Bono C M, Schwab J H. Predicting prolonged opioid prescriptions in opioid-naïve lumbar spine surgery patients. Spine J 2020b; 20(6): 888-95.

Karnuta J M, Navarro S M, Haeberle H S, Billow D G, Krebs V E, Ramkumar P N. Bundled care for hip fractures: a machine learning approach to an untenable patient-specific payment model. J Orthop Trauma 2019; 33(7): 324-30.

Karnuta J M, Golubovsky J L, Haeberle H S, Rajan P V, Navarro S M, Kamath A F, Schaffer J L, Krebs V E, Pelle D W, Ramkumar P N. Can a machine learning model accurately predict patient resource utilization following lumbar spinal fusion? Spine J 2020; 20(3): 329-36.

Katakam A, Karhade A V, Schwab J H, Chen A F, Bedair H S. Development and validation of machine learning algorithms for postoperative opioid prescriptions after TKA. J Orthop 2020; 22: 95-9.

Khan O, Badhiwala J H, Witiw C D, Wilson J R, Fehlings M G. Machine learning algorithms for prediction of health-related quality-of-life after surgery for mild degenerative cervical myelopathy. Spine J 2020: S1529-9430(20)30047-4. Online ahead of print.

Kim J S, Arvind V, Oermann E K, Kaji D, Ranson W, Ukogu C, Hussain A K, Caridi J, Cho S K. Predicting surgical complications in patients undergoing elective adult spinal deformity procedures using machine learning. Spine Deform 2018a; 6(6): 762-70.

Kim J S, Merrill R K, Arvind V, Kaji D, Pasik S D, Nwachukwu C C, Vargas L, Osman N S, Oermann E K, Caridi J M, Cho S K. Examining the ability of artificial neural networks machine learning models to accurately predict complications following posterior lumbar spine fusion. Spine (Phila Pa 1976) 2018b; 43(12): 853-60.

Kukar M, Kononenko I, Silvester T. Machine learning in prognosis of the femoral neck fracture recovery. Artif Intell Med 1996; 8(5): 431-51.

Kumar V, Roche C, Overman S, Simovitch R, Flurin P H, Wright T, Zuckerman J, Routman H, Teredesai A. What is the accuracy of three different machine learning techniques to predict clinical outcomes after shoulder arthroplasty? Clin Orthop Relat Res 2020; 478(10): 2351-63.

Kunze K N, Karhade A V. Sadauskas A J, Schwab J H, Levine B R. Development of machine learning algorithms to predict clinically meaningful improvement for the patient-reported health state after total hip arthroplasty. J Arthroplasty 2020;3 5(8): 2119-23.

Lin C-C, Ou Y-K, Chen S-H, Liu Y-C, Lin J. Comparison of artificial neural network and logistic regression models for predicting mortality in elderly patients with hip fracture. Injury 2010; 41(8): 869-73.

Lungu E, Vendittoli P A, Desmeules F. Identification of patients with suboptimal results after hip arthroplasty: development of a preliminary prediction algorithm. BMC Musculoskelet Disord. BMC Musculoskeletal Disorders; 2015; 16(1): 1-10.

Martini M L, Neifert S N, Oermann E K, Gal J, Rajan K, Nistal D A, Caridi J M. Machine learning with feature domains elucidates candidate drivers of hospital readmission following spine surgery in a large single-center patient cohort. Neurosurgery 2020; 87(4): E500-10.

Merali Z G, Witiw C D, Badhiwala J H, Wilson J R, Fehlings M G. Using a machine learning approach to predict outcome after surgery for degenerative cervical myelopathy. PLoS One 2019; 14(4): e0215133.

Merrill R K, Ferrandino R M, Hoffman R, Shaffer G W, Ndu A. Machine learning accurately predicts short-term outcomes following open reduction and internal fixation of ankle fractures. J Foot Ankle Surg 2019; 58(3): 410-6.

Nwachukwu B U, Beck E C, Lee E K, Cancienne J M, Waterman B R, Paul K, Nho S J. Application of machine learning for predicting clinically meaningful outcome after arthroscopic femoroacetabular impingement surgery. Am J Sports Med 2020; 48(2): 415–23.

Ogink P T, Karhade A V, Thio Q C B S, Hershman S H, Cha T D, Bono C M, Schwab J H. Development of a machine learning algorithm predicting discharge placement after surgery for spondylolisthesis. Eur Spine J 2019a; 28(8): 1775-82.

Ogink P T, Karhade A V, Thio Q C B S, Gormley W B, Oner F C, Verlaan J J, Schwab J H. Predicting discharge placement after elective surgery for lumbar spinal stenosis using machine learning methods. Eur Spine J 2019b; 28(6): 1433-40.

Ottenbacher K J, Linn R T, Smith P M, Illig S B, Mancuso M, Granger C V. Comparison of logistic regression and neural network analysis applied to predicting living setting after hip fracture. Ann Epidemiol 2004; 14(8): 551-9.

Paulino Pereira N R, Janssen S J, van Dijk E, Harris M B, Hornicek F J, Ferrone M L, Schwab J H. Development of a prognostic survival algorithm for patients with metastatic spine disease. J Bone Joint Surg Am 2016; 98(21): 1767-76.

Pua Y H, Kang H, Thumboo J, Clark R A, Chew E SX , Poon C L L, Chong H C, Yeo S J. Machine learning methods are comparable to logistic regression techniques in predicting severe walking limitation following total knee arthroplasty. Knee Surg Sport Traumatol Arthrosc 2020; 28(10): 3207-16.

Ramkumar P N, Navarro S M, Haeberle H S, Karnuta J M, Mont M A, Iannotti J P, Patterson B M, Krebs V E. Development and validation of a machine learning algorithm after primary total hip arthroplasty: applications to length of stay and payment models. J Arthroplasty 2019; 34(4): 632-7.

Scheer J K, Smith J S, Schwab F, Lafage V, Shaffrey C I, Bess S, Daniels A H, Hart R A, Protopsaltis T S, Mundis G MJ , Sciubba D M, Ailon T, Burton D C, Klineberg E, Ames C P. Development of a preoperative predictive model for major complications following adult spinal deformity surgery. J Neurosurg Spine 2017; 26(6): 736-43.

Schwartz M H, Ward R E, Macwilliam C, Verner J J. Using neural networks to identify patients unlikely to achieve a reduction in bodily pain after total hip replacement surgery. Med Care 1997; 35(10): 1020-30.

Shi L, Wang X C, Wang Y S. Artificial neural network models for predicting 1-year mortality in elderly patients with intertrochanteric fractures in China. Braz J Med Biol Res 2013; 46(11): 993-9.

Siccoli A, de Wispelaere M P, Schröder M L, Staartjes V E. Machine learning-based preoperative predictive analytics for lumbar spinal stenosis. Neurosurg Focus 2019; 46(5): E5.

Thio Q C B S, Karhade A V, Ogink P T, Bramer J AM , Ferrone M L, Calderón S L, Raskin K A, Schwab J H. Development and internal validation of machine learning algorithms for preoperative survival prediction of extremity metastatic disease. Clin Orthop Relat Res 2020; 478(2): 322-33.

Wu H-Y, Gong C-S A, Lin S-P, Chang K-Y, Tsou M-Y, Ting C-K. Predicting postoperative vomiting among orthopedic patients receiving patient-controlled epidural analgesia using SVM and LR. Sci Rep 2016; 6: 27041.

Zhang Y, Fatemi P, Medress Z, Azad T D, Veeravagu A, Desai A, Ratliff J K. A predictive-modeling based screening tool for prolonged opioid use after surgical management of low back and lower extremity pain. Spine J 2020a; 20(8): 1184-95.

Zhang Y, Huang L, Liu Y, Chen Q, Li X, Hu J. Prediction of mortality at one year after surgery for pertrochanteric fracture in the elderly via a Bayesian belief network. Injury 2020b; 51(2): 407-13.
